# A systematic review of occupational exposure to coal dust and the risk of interstitial lung diseases

**DOI:** 10.1080/20018525.2017.1264711

**Published:** 2017-01-03

**Authors:** Christiane Beer, Henrik A. Kolstad, Klaus Søndergaard, Elisabeth Bendstrup, Dick Heederik, Karen E. Olsen, Øyvind Omland, Edward Petsonk, Torben Sigsgaard, David L. Sherson, Vivi Schlünssen

**Affiliations:** ^a^ Department of Public Health, Section for Environment, Occupation and Health, Danish Ramazzini Centre, Aarhus University, Aarhus, Denmark; ^b^ Department of Occupational Medicine, Danish Ramazzini Centre, Aarhus University Hospital, Aarhus, Denmark; ^c^ Department of Rheumatology, Aarhus University Hospital, Aarhus, Denmark; ^d^ Department of Respiratory Diseases and Allergy, Aarhus University Hospital, Aarhus, Denmark; ^e^ Institute for Risk Assessment Science, Utrecht University, Utrecht, The Netherlands; ^f^ Department of Pathology, Odense University Hospital, Odense, Denmark; ^g^ Department of Occupational Medicine, Danish Ramazzini Centre, Aalborg University Hospital, Aalborg, Denmark; ^h^ Department of Medicine, West Virginia University School of Medicine, Morgantown, WV, USA; ^i^ Department of Occupational and Environmental Medicine, University of Southern Denmark, Odense, Denmark; ^j^ Department of Pulmonary Medicine, University of Southern Denmark, Odense, Denmark; ^k^ National Research Centre for the Working Environment, Copenhagen, Denmark

**Keywords:** Mining, coal dust, coal workers’ pneumoconiosis, interstitial lung disease, non-quartz coal dust, quartz

## Abstract

**Objective**: Exposure to coal dust can cause interstitial lung disease (ILD), but whether this is due to pure coal or to the contents of quartz in coal is less clear. Here, we systematically reviewed the relation between ‘pure coal’ and ILD.

**Methods**: In a systematic review based on PRISMA criteria 2945 articles were identified. Strict eligibility criteria, which evaluated the ‘pure coal effect’, led to the inclusion of only nine studies.

**Results**: Among these nine studies six studies indicated an independent effect of the non-quartz part of coal on the development and progression of ILD, two did not demonstrate an effect and one was inconclusive.

**Conclusions**: Although an independent effect of non-quartz coal dust on the development of ILD is supported, due to methodological limitations the evidence is limited and further evidence is needed.

## Introduction

Despite the increasing demand for alternative energy sources, coal is still an important energy source worldwide. Around 30% of global energy needs are covered by coal generating 41% of the world’s electricity. It is used in 70% of the world’s steel production (http://www.worldcoal.org/resources/coal-statistics/). Based on statistics from the World Coal Association the total world coal production reached a record level of 7831 Mt in 2012, which is a 2.9% increase in comparison to 2011, suggesting an increasing coal demand. Development of lung diseases caused by exposure to coal dust and coal impurities like quartz and iron has been studied over the last century. It is well documented that exposure to coal dust can cause respiratory disorders.[1–3] The major focus has been on interstitial lung disease (ILD), although chronic airflow obstruction is also widely recognized as a health outcome related to coalmine dust exposures.[4,5] ILD comprises a group of uncommon and often severe lung diseases characterized by pulmonary fibrosis. Quartz exposure is a well-documented cause of ILD named silicosis. Other causal risk factors for ILD are asbestos (asbestosis), specific drugs, radiation, connective tissue disease as well as biological and chemical agents (allergic alveolitis).[6] The prognosis for most cases of ILD is poor [7] and lung transplantation is the only effective cure.

Lung diseases associated to coalmine dust are named according to the exposure suspected to cause the disease, i.e. coal workers’ pneumoconiosis (CWP), silicosis and mixed dust pneumoconiosis. Anthracosis is used both as a synonym for CWP and as massive carbon deposits seen in the lung at autopsy. Recently, dust-related diffuse fibrosis (DDF) has also been described.[3] Risk factors for CWP are primarily related to airborne dust concentration, exposure duration, and coal characteristics, e.g. coal rank, quartz and iron content. However, genetic predisposition might also play a role.[8,9]

Although the association between coal mine dust exposure and lung disease has been investigated for decades it is still not clear what components of the coal dust are actually responsible for disease development. Previously there has been a particular interest in quartz as an important fibrogenic component.[10] However, some epidemiological and experimental studies suggest only a minor fibrogenic role for quartz among coal dust exposed workers.[8] Lung pathology studies among workers exposed to coal dusts with little or no silica have documented extensive CWP.[3] An inverse relationship between quartz and CWP has also been described,[11] and higher coal rank with increased iron may correlate with CWP risk.[12]

There are presently no systematic reviews on the non-quartz part of coal and development of lung diseases, and the aim of our study was to systematically review what is known about the relation between exposure to the non-quartz part of coal dust and ILD.

## Methods

### Search strategy

This systematic review is based on PRISMA (preferred reporting items for systematic reviews and meta-analyses) criteria,[13] a revision of the QUOROM (quality of reporting of meta-analysis) guidelines.[14]

The following international databases were used for the literature search performed April 2014 and updated in January 2016: the National Library of Medicine (PubMed), Embase, and the Cochrane Library. In addition, searches were performed in the Scandinavian databases bibliotek.dk and SveMed+. The literature search was performed by CB based on the search steps shown in Supplementary Table A. Depending on the database, we included papers published in English, Danish, Swedish, and Norwegian. All articles were reviewed independently by two authors. Both reviewers had to agree on an article before it was included for data extraction. The selection of articles was based on the following eligibility criteria: epidemiologic peer-reviewed studies on relevant exposure and outcome including case-control, cross-sectional and follow-up studies with an external control group or exposure contrast in the exposed group. As our objective was to investigate the relation between the non-quartz part of coal exposure and ILD, it was a prerequisite for inclusion of a study that the quartz content was taken into consideration. Specifically, articles were only included if both the mineral fraction and the pure coal fraction of dust was given. Furthermore mutual adjustment of the ‘pure coal effect’ for the ‘mineral dust effect’ in a multiple regression type of analysis or a stratified analysis which allows evaluation of the ‘pure coal effect’ was required for inclusion.

ILD (including pneumoconiosis and CWP) was the relevant outcome. As the retrieved literature covered several decades, the diagnostic criteria varied over time. The ILD diagnosis was either clinical or based on death certificates or pathology. The clinical diagnosis of ILD was based on X-ray films, lung function or both.

The literature search resulted in a total of 2665 unique articles. Based on title or abstract 2440 articles were excluded. After reading of 225 full papers, eight articles were regarded suitable for data extraction. For details see Supplementary Table A.

An additional search for reviews and meta-analysis on coal exposure and ILD was performed. The search term was ‘coal’ and the search was limited to systematic reviews, meta-analysis, and Cochrane-reviews in English that were officially categorized as these article types by the used databases. Based on snowball search, where the references in the eight original articles and the 486 identified reviews/meta-analysis were screened for relevant studies one additional article was identified. Thus a total of nine studies were included.

Data extracted from the articles are presented in Table 1 and include publication year, country where the study was performed, study design, study period and study population, number of exposed and controls, age and age range, exposure levels and duration, how the exposure assessment was performed, the presence or absence of exposure-response analysis, outcome, and covariates accounted for.

**Table 1.** Characteristics and main results of nine epidemiologic studies of lung function and pneumoconiosis among coal exposed individuals, with consideration of the quartz content of the coal.

Author, year, country [ref.]	Study design (study period)	Study **population: exposed** /control	Age (range)	Exposure assessment	Exposure levels and duration	Exposure-response analysis	Outcome (measure)	Covariates **accounted** for	Result
Casswell 1970 UK [15]	Cross sectional study among deceased coal miners	98/0*	No data	Measurement of recovered lung dust; X-ray diffractometry or fluorescence analysis of mineral matter, including quartz and iron	No data	No	Full sized chest radiograph: modelled mean of 11 readings between 0/– and 3/4. ILO classification 1959.	Quartz	Both coal dust and mineral dust increased in the lungs in relation to radiological score: Coal: β = 0.126 (SD 0.01). Mineral: β = 0.533 (SD 0.04).Correlation between mineral and quartz: r = 0.96.
Seaton 1981 UK [16]	Nested case control from one British colliery. Same mine as in Buchanan et al. 2003	21 cases; 21 controls	No data	Thermal precipitator and MRI gravimetric dust sampling from 1954 to 1978. Cumulative exp. 1974–1978 by occupational group times duration within each group.	Cases: resp. dust 11,478 mg h m^–3^ Controls: resp. dust 7173 mg h m^–3^ Range exp. duration: ~30,000–70,000 h	Yes	Full sized posterior-anterior chest radiograph: One reader: 1+ change in opacities 1974 to 1978. ILO classification 1972.	Age matched controls, quartz	Cases vs. controls: % coal in resp. dust: 57 vs. 63%, *p* < 0.05 % quartz in resp. dust: 13 vs. 8%, *p* < 0.05
Hurley 1982 UK [17]	Cross sectional study, 10 British coal mines	2600/0*	52 (35–65)	Thermal precipitator and MRI gravimetric dust sampling from 1954 to 1978. Cumulative exp. by occupational group times duration within each group.	Mean resp. dust 183 g h m^–3^ Mean quartz 8.8 g h m^–3^ Mean duration 33 years	Yes	Full sized posterior-anterior chest radiograph, mean of five readers: Opacities 2/1+ (ILO classification 1968).	Quartz	Strong effect of cumulative dust exp. when stratified by cumulative quartz exp. Coal dust has an effect on opacities that cannot be explained by quartz or ash. More rapid progression at very high quartz content
Jacobsen 1982 UK [18]	Nested case control study among 4333 miners from 10 British coal mines. Same mines as in Hurley et al. 1982	45 cases; 41 controls	Cases 43 Controls 44	Thermal precipitator and MRI gravimetric dust sampling from 1954 to 1978. Cumulative exp. by occupational group times duration within each group.	Cases: resp. dust 43 g h m^–3^ Controls: resp. dust 38 g h m^–3^ Exp. duration >10 years	No	Full sized posterior-anterior chest radiograph: Change: 2+ step (ILO classification 1968)	Smoking, quartz	Mean difference cases vs. controls: Resp. mixed coal dust 5.2 g h m^–3^ Quartz 0.75 g h m^–3^ Quartz % of mixed coal dust 1% Quartz-related unusually rapid progression of opacities
Ruckley 1984 UK [19]	Cross sectional study among deceased coal miners	261/0*	No data	Measurement of recovered lung dust and infrared spectrophotometry of residual ash, including quartz	No data	No	Full size posterior-anterior chest radiograph: opacities ILO classification 1971). Three gradings of pathological findings: O; 1 (< 1.5 mm); 2 (1.5 –3 mm); 3 (> 3 mm). > 10 mm excluded.	Quartz	Low rank coal: the increase in profusion was most closely related to the ash component of the total dust High rank coal: both coal dust and ash increased in the lungs in relation to radiological profusion
Douglas 1986 UK [20]	Cross sectional study among deceased coal miners	430/0* Autopsied lung pairs. (1972–1974).	No data	Thermal precipitator and MRI gravimetric dust sampling from 1954 to 1980. Average level by occupational group times duration within each group.	Resp. dust 136.9–303.1 g h m^–3^ Carbon 81.1–94% Quartz 2.8–5.4% Exp. duration 27–44 years	Yes	Three gradings of pathological findings: 1: < 1 mm; 1–9 mm and 10+ mm lesions	Quartz	Increasing lesions with increased whole dust and ash deposits in the lungs. Not possible to evaluate the quartz effect properly.
Morfeld 1997 Germany [21]	Follow up study. Mean follow-up time 9.3 years, three German coal mines	5778/0*	No data	43,842 gravimetric dust sampling, individual and area measurements.	Mean resp. dust 1.8 mg m^–3^ Mean quartz 0.18 mg m^–3^ Between 1.7 and 12.5% quartz Mean exp. duration 21.1 years	Yes	Full and small sized anterior-posterior chest radiograph: Opacities 0/1+ (ILO classification 1980)	Quartz	1 mg m^–3^ vs. 3 mg m^–3^ resp. dust adjusted for quartz dust: RR = 0.98 (*p* = 0.98) No relationship between coal dust and abnormal radiological finding. Strong ‘mine’ effect not explained by dust or quartz concentration
Buchanan 2003 UK [22]	Cross-sectional study, 1990–1991, one British colliery. One of the 10 mines included in Hurley 1982	371/0*	50–74	Thermal precipitator and MRI gravimetric dust sampling from 1954 to 1980. Average level by occupational group times duration within each group.	Mean non-quartz resp. dust 53.1 g h m^–3^ Mean quartz 4.48 g h m^–3^ Mean exp. duration 8000 h	Yes	Full size posterior-anterior chest radiograph: Opacities 2/1+ (ILO classification 1980)	Smoking, age, quartz	OR, resp. dust g h m^–3^ pre-1964 adjusted for quartz: 0.996 (0.971–1.022) OR, resp. dust g h m^–3^ post-1964 adjusted for quartz: 1.025 (0.974–1.079) Absolute risk for 15 years exp. to non-quartz resp. dust: 0.8%
Graber 2014 USA [23]	Follow-up mortality study 1969–2007, underground coal mine workers. Mean follow-up time 20.8 years	8829/0*	Mean 45 at start of follow-up.	Mine safety and health administration compliance data from 1982–2002. Gravimetric sampling. Average level by occupational group times duration within each group.	Mean coal dust 64.6 mg m^–3^ year Quartz 2.6 mg m^–3^ year	Yes	Pneumoconiosis from death certificates Full size anterior-posterior chest radiograph (ILO classification 1980)	Smoking, age, coal rank, race, calendar year, quartz	Cox proportional hazard analysis. Resp. dust HR adjusted for quartz between 1.17 and 2.58 depending on mine, highest for hard coal mines. Interaction between dust and mine. Quartz HR adjusted for resp. dust: 1.33 (0.94–1.90)

*No internal control group; PMF – Progressive massive fibrosis; FEV1 – forced expiratory volume in one second; FVC – forced vital capacity; FEV1/FVC – ratio FEV1/FVC; ILO – International Labour Organization; RR – relative risk; OR – odds ratio; Resp. – respirable; Exp. – exposure.

## Results

### Description of included papers

The results of the included studies are summarized in Table 1. Seven studies were based in the UK;[15–20,22] one was performed in Germany [21] and one in USA.[23] The included studies originated mostly from open cohorts of mineworkers employed in specific mines followed several years with exposure measurements and chest X-rays. The performed analyses were either cross-sectional,[15,17–20,22] longitudinal with average follow-up periods between 9.3 [21] and 21 years [23] or nested case control studies.[16,18] The study populations varied between 21 [16] and 8829 [21,23] (median number of participants 371). The mean age was between 43 years [18] and 52 years [17,18,24] and the age range in the studies was between 35 [17] and 74 years.[22] In most of the studies in Table 1 neither the mean age nor age range was specified.[15,16,19–21] The study population in all studies was solely males.

All follow-up and cross-sectional studies compared exposure levels within coal miners and did not have external exposure groups.[15,17,19–23]

The studies by Seaton et al. [16] and Buchanan et al. [22] investigated the same mine; however, in different subpopulations and with different study designs and set-ups. The same was true for the studies by Jacobsen et al. [17] and Hurley et al. [18]. Furthermore, a study by Morfeld et al. [25] has been excluded as a subset of the study population was already included in [21].

### Exposure measurements

The exposure measurements were performed with gravimetric personal or area dust sampling in all studies and were given as average mean exposure mg m^–^^3^ or average cumulative exposure g h m^–3^ (gram per cubic metre (g m^–3^) multiplied by duration of exposure in hours (h)) for coalmine dust and quartz, respectively (Table 1). The quartz content varied between 1% [18] and 13%.[16] The exposure duration was between >10 years [18] and up to 44 years.[20] In two studies, the exposure duration was not stated.[15,19] The study population described by Seaton et al. [16] was exposed between 30,000 and 70,000 h and in the study of Buchanan et al. [22] the exposed individuals were on average exposed to coalmine dust for 8000 h. Cumulative exposure to respirable dust and respirable quartz was in most studies assessed from work duration in specific tasks or jobs times the average measured exposure in each of the tasks/jobs.[15–23] In two studies among deceased coal miners, exposure was assessed as coal dust and quartz content in the lung tissue using infrared spectrophotometry, X-ray diffractometry or fluorescence analysis.[15,19] Covariates that were accounted for included quartz (all studies); age,[16,22,23] and smoking.[18,22,23] Except for three studies,[15,18,19] exposure–response analyses were performed in all studies.

### Adjustment for quartz content

The quartz dust content was taken into consideration in different ways. Buchanan et al. [22] adjusted for cumulative quartz exposure using general exposure index (GEI) models based on the mean quartz concentration for each period, the number of hours worked, and the time from exposure period to follow-up. Work hours and periods were collected from exposure history questionnaires.[22] A detailed analysis of the coal dust samples by infrared spectro-photometry using the KBr disc methods were performed by Seaton et al. [16] and Jacobsen et al. [18]. They report precise information on the carbon and quartz content in the lungs that allowed for a direct comparison of X-ray progression index and carbon and quartz content.[16,18] In their study from 1997, Morfeld et al. [21] used Cox regression modelling with time-dependent covariates including quartz to estimate the relative risk of opacities in relation to the non-quartz component. Hurley et al. [17] used a micro-infrared technique and infrared spectroscopy for samples that were taken during the last 10 years of the study. The assessment of the quartz concentration by Douglas et al. [19] and Ruckley et al. [20] was done after pathological examination on autopsies and the dust composition (coal, quartz, etc.) was determined on samples of whole dried lung tissue by calculating the weight loss after ashing (coal) and infrared spectrophotometry (quartz), or in the case of Casswell et al. [15] by using X-ray diffractometry and fluorescence analysis. Graber et al. [23] used data from the Mine Safety and Health Administration to estimate the amount of respirable quartz dust exposure for job groups and assigned an individual cumulative quartz exposure estimate to each study participant.

### Diagnostic criteria

The studies were performed between 1981 and 2010, covering nearly 30 years with different diagnostic criteria. Except for Douglas et al. [20] who based the diagnosis on autopsies, the evaluation of lung opacities and diagnosis of ILD was based on the most recent International Labour Organization (ILO) classification scheme that was valid at the time of publishing.[15–19,21–23] The guidelines were updated in 2011.[26] Most versions define four categories (0, 1, 2 and 3) that are based on profusion of small opacities, where 0 indicates an absence of opacities or opacities less than for category 1. There are 12 subcategories. Examples of categorization are 0/1, 1/0, 1/1, etc., where the first figure denotes the category which best matches the X-ray image and the second figure indicates the subcategory that has been considered as an alternative. The studies included between one reader,[16] up to the mean of 11 readers [15] evaluating development or progression of opacities. In one study consensus was reached between two to three readers,[21] and one study defined progression if at least three out of five readers agreed on this.[18]

### Associations between coal dust and ILD

Buchanan et al. [22] found no significant association between cumulative respirable dust and opacities 2/1+ after adjustment for quartz. However, the absolute risk for opacities 2/1+ after 15 years’ exposure to non-quartz respirable dust was 0.8%. Seaton et al. [16] found that cases (change in opacities progression of at least 1+) had been exposed to higher % quartz but lower % coal and concluded that quartz might be an important factor in the development and rapid progression of opacities. Jacobsen et al. [18] came to the same conclusion. They reported a mean difference between cases (cases defined as opacities progression of at least two categories) and controls of respirable mixed dust of 5.2 g h m^–3^ and quartz of 0.75 g h m^–3^. The mean difference of quartz % in mixed dust was 1%. However, they also found that not all cases with progressive massive fibrosis had higher exposures to quartz than corresponding controls.[18] Morfeld et al. [21] did not find a relationship between cumulative coalmine dust exposure and abnormal radiological findings. However, they report a strong mine effect on the development of opacities 1+ that could not be explained by dust or quartz concentration. Hurley et al. [17] reported a strong effect of cumulative dust exposure on the occurrence of opacities 2/1 even after stratifying by cumulative quartz exposure, and the effect of dust on the opacities could not be explained by quartz or ash (mineral part of the coal). However, high quartz concentrations had an effect on rapid progression of opacities. Douglas et al. [20] found that lung lesions increased with whole dust and ash deposits; however, it was not possible to evaluate the quartz effect properly. For exposure to low rank coal Ruckley et al. [18] showed that the increase in radiologic profusions was most closely related to the ash component, whereas for exposure to high rank coal, that is bituminous and anthracite coals with a high carbon content, both coal dust and ash content were each associated to radiological profusions. This is in agreement with the findings of Casswell et al. [15] who showed that both coal and mineral dust in lung tissue were associated to the radiological findings. Graber et al. [23] found hazard ratios between 1.17 and 2.58 for respirable coal dust and pneumoconiosis mortality after adjustment for quartz. In addition, the effect depended on the mine and was highest for hard coal mines.

## Discussion

Among nine studies six indicated an independent effect of non-quartz/non-mineral coal dust on the development of ILD. It is evident that major methodological limitations are present in most studies.

The objective of this review was to investigate specifically which role the non-quartz part of coal dust plays for the risk of ILD, and articles were only included when either information on the quartz level was available or when the data were adjusted for the quartz fraction of the coal. Several authors have questioned the importance of quartz in coal dust, as quartz has been found to be a minor contributor to ILD among coal dust exposed workers.[8,27] There are studies where quartz could not be attributed either to CWP or progressive massive fibrosis [28–33] unless the quartz concentration exceeds 10%. This was confirmed in *in vivo* animal studies of rats where coal dust was supplemented with different concentrations of quartz.[8,34–36]

Three studies aimed at investigating the effect of the quartz on the occurrence of opacities and thereby indirectly stating the effect of the non-quartz part of the mixed coalmine dust.[16–18] In Hurley et al. [17], a large study with a high quality exposure assessment, they excluded an effect of ash. As ash is the mineral part of coalmine dust they thereby stated that the carbon part was responsible for the development of opacities. The study by Jacobsen et al. [18] investigated progression of opacities. The authors concluded that rapid progression of opacities was due to high quartz content but they also found that exposure to quartz was not the sole factor responsible for progressive massive fibrosis.[18]

When quartz was accounted for in Buchanan et al. [22] the predicted risk to develop opacities 2/1+ after 15 years exposure was still 0.8%, and also Graber et al. [23] found a hazard ratio for cumulative respirable coal dust after adjustment for quartz between 1.17 and 2.58, depending on the mine. Furthermore, Ruckley et al. [15] and Casswell et al. [19] showed an effect of the carbon part of the mixed coalmine dust on the appearance of opacities. Both studies were performed directly on the lungs of deceased coal miners and measured the characteristics and concentrations of the recovered lung dust. Ruckley et al. [19] found that both the coal dust and the ash concentration increased in the lungs in relation to radiological profusion. However, this effect was only seen for high rank coal. The study of Casswell et al. [15] showed as well that the coal dust but also the mineral dust increased in the lungs in relation to the radiological score. The strength of both studies is that the depositions of the mixed coalmine dust are directly measured. However, both studies did not give any details on the exposure level, exposure duration and did not account for any confounders. Seaton et al. [16] and Morfeld et al. [21] did not show a relation between the non-quartz part of coalmine dust and the appearance of opacities. In Morfeld et al. [21] they used a different methodology using small size films that are not compatible with ILO standards. From the study performed by Douglas et al. [20] it was not possible to evaluate the effect of the non-quartz part of the mixed coal mine dust. However, an increasing amount of mixed coal dust and ash deposits increased the observed lesions in the lungs.

There are no systematic differences in the studies supporting or refuting an independent effect of non-quartz/non-mineral coal dust on development of ILD with regard to study design, or exposure assessment. Morfeld et al. [21] was the only study who partly used small size films, and this might have underestimated the number of opacities and could explain the strong mine effect reported by the authors. The levels were substantially higher in the positive studies, roughly 150,000 mg h m^–3^ [17,22,23] compared to the negative studies, roughly 30,000 mg h m^–^^3^.[16,18] The only three studies that adjusted for age, smoking and quartz supported an independent effect of non-quartz/non-mineral coal dust on development of ILD.[18,22,23]

There are several limitations in the included studies. Most of the analyses were cross-sectional and information about dropout rates was not given.[15,17–20,22] Due to the comprehensive objective quantitative exposure assessment in most studies, information bias is probably not an issue. However, selection out of the cohort is likely and its effect on the measures of association presented in the different studies cannot be properly evaluated. With increasing cumulative coal dust exposures, miners may experience increasing respiratory symptoms, FEV_1_ (forced expiratory volume in one second) losses, and declines in diffusing capacity for carbon monoxide [35] and therefore might be prone to leave the coal mining industry.

There were no studies addressing the pure carbon part of coal dust. Different methods have been used in all the studies to take quartz into account by modelling, adjustment or by simple subtraction. However, it remains a drawback that studies with populations exposed to pure carbon alone are not available.

All study populations consisted solely of males. Therefore, this review provides no information about particular risks of these exposures for females caused by possible gender specific susceptibility as smaller airway calibres [37] or hormonal factors.[38] Another limitation is the exclusion of papers not published in English or Nordic language journals. Parts of the world have extensive coal mining activities, for example Germany, South America and China, and publications from those countries in their native languages are not included in this review. One can imagine that positive studies are more easily published in high ranking English journals, leaving the negative studies to the native language journals. At least for controlled trials it does not seem to impact the results of meta-analysis substantially,[39] but we cannot be sure that leaving those out from the review may have impacted the overall result and limited the external general validity of this systematic review.

According to the followed PRIMA criteria [13] two persons after an initial independent selection have to agree on included and excluded papers. However, the majority of papers were excluded based on screening of titles and abstract. Given the large amount of papers identified in the first place (2941) it is possible that some relevant papers were not included. The procedure with two persons limits this risk, but does not completely remove it. We do not think this possible false discarding of one or more papers depended on the result of the study, and therefore we do not think it has seriously biased our overall conclusions in the review.

Smoking was only taken into account in a few studies.[18,22,23] Smoking is regarded a risk factor for most subtypes of ILD, including idiopathic pulmonary fibrosis,[40,41] also for parenchymal opacities (>1/0), and it is suggested that smoking is a true risk factor as well as an effect modifier.[42] As most studies were adjusted for neither smoking nor age or person-year the results may in general overestimate the effect of coal dust as cumulative exposure to coal dust and cumulative exposure to smoking is probable correlated.

Another limitation might be associated with the diagnosis of ILD, in the included papers stated as CWP. CWP and silicosis are subtypes of ILD defined by the type of exposure (coal and quartz, respectively), which is an obstacle when aiming to investigate these associations. All included papers, though, have used these terms, and therefore they are also used in this review. The identification and diagnosis of CWP is traditionally based on exposure history and respiratory symptoms in combination with radiological changes (opacities) based on routine chest radiographs (including digital radiographs) excluding other causes of fibrosis.[3,43] The presence and the severity of CWP is classified using the ILO classification system where CWP is classified according to the number, size, and shape of small opacities using standard reference films developed by the ILO.[44] The ILO criteria used differed between the studies, and included between one reader [16] and up to 11 readers [15] with a variable agreement. In addition, a limitation of film-based chest radiographs is their low sensitivity. Pathologic abnormalities have to be moderate to severe before they can be detected with certainty in comparison to current digital radiological techniques. In a current clinical setting for diagnosing ILD, lung function test and radiological findings are combined with high-resolution computed tomography and in uncertain cases lung biopsy. Furthermore, progressive massive fibrosis might be confused with carcinoma, tuberculosis, or bacterial infectious lesions,[44] and this might affect the findings in the included studies.

ILD in coal dust exposed workers may be complex. Lesions typical for silicosis were found in conjunction with CWP in 8% of autopsied lungs from coal workers employed in modern US conditions, and in 28% of lungs from those who had worked before regulation of dust exposure.[45] Classical chest radiography is not able to distinguish between CWP and silicosis, and tissue examinations are preferable for a reliable determination of the diagnosis, as done in the studies by Ruckley et al. [15] and Casswell et al. [19], which both showed an effect of the carbon part of the mixed coal mine dust on the appearance of opacities.

It is biologically plausible that the pure carbon part of coal dust can induce lung opacities. Important factors for the uptake and fate of coal dust particles in the lungs include the chemical and morphological properties of the dust particles as well as lung volume, breathing rate and depth.[46] The coal dust particles might produce ROS (reactive oxygen species) directly at the particle surface through surface radicals and ions. Increased formation of ROS has been associated with a number of diseases and has been shown to induce damage to cell membranes, increased lipid peroxidation, oxidation of proteins, and DNA damage.[46]

Dust from anthracite coal has been associated with higher cytotoxicity and pathogenicity than dust from bituminous coal. This has been explained by the higher amount of free radicals at the surface of anthracite coal dust particles.[45,47,48] In addition, the porous surface of the coal dust particles leads to a large surface area where compounds like benzene, phenol, and methylene that are present in the coal-mining atmosphere can be adsorbed.[45] The stronger toxicity of high rank coal was supported by Graber et al. [19] and Ruckley et al. [23]. This is in agreement with other studies that found higher rates of CWP for anthracite coal compared to bituminous coal.[49,50] Graber et al. [19] and Ruckley et al. [23] also found this higher effect for the non-quartz part of the mixed coal mine dust in high rank coal mines, suggesting that the higher amount of free radicals at the surface of anthracite coal dust particles plays an important role in the toxicity of mixed coal mine dust. However, in a recent review on several Chinese studies Mo et al. [51] showed similar CWP risk for anthracite (5.38; 95% CI 2.11, 10.04) and bituminous coal (5.88; 95% CI 2.21, 11.16).

Based on the six included studies with available data (five from UK and one from USA), the cumulative coal mine dust exposure level was roughly calculated to 100,000 mg h m^–3^ with exposure duration between 3.8 and 33 years.[16–18,20,22,23] The levels were significantly higher in the positive studies compared to the negative studies. Most studies revealed a positive dose–response relation between coal mine dust and ILD,[17,20,22,23] but it was not possible to rule out dose–response relations for the pure coal fraction of the dust. There is clear indications of a declining trend for coalmine dust in both the UK from 1970–1979 [52] and in Germany from 1984–1998.[25] In Buchanan et al. [22] they included measurements from 1954–1980 and found that after 15% years exposure with non-quartz-respirable dust 0.8% had developed ILD. So all together, given a true causal association between pure coal dust and ILD, we judge the absolute risk to be low.

## Conclusion

Most of the included studies suggest an independent effect of non-quartz /non-mineral coal dust on the development of ILD. The majority of the analyses were cross-sectional and in all studies, quartz exposure was present to some degree, whereas smoking and age was rarely taken into account. The diagnosis of ILD was not based on current clinical definitions. Of note, none of the studies included women in their investigations, so no conclusions can be made for women exposed to coal dust.

We find that the degree of evidence of a causal association between pure coal dust exposure and ILD is limited. In order to strengthen the evidence, well conducted follow-up analyses on workers exposed to coal dust with no or very low mineral content are needed.

## Supplementary Material

Supplementary Material

## References

[CIT0001] Collis EL. (1931). Recent views on pneumonoconioses. Proc R Soc Med.

[CIT0002] Trotter RS (1903). The so-called anthracosis and phthisis in coal miners. Br Med J.

[CIT0003] Petsonk EL, Rose C, Cohen R (2013). Coal mine dust lung disease. New lessons from an old exposure. Am J Respir Crit Care Med.

[CIT0004] Coggon D, Newman Taylor A (1998). Coal mining and chronic obstructive pulmonary disease: a review of the evidence. Thorax.

[CIT0005] The National Institute for Occupational Safety and Health (NIOSH) (1995). Criteria for a recommended standard: occupational exposure to respirable coal mine dust.

[CIT0006] Demedts M, Wells AU, Anto JM (2001). Interstitial lung diseases: an epidemiological overview. Eur Respir J Suppl.

[CIT0007] Hyldgaard C, Hilberg O, Muller A (2014). A cohort study of interstitial lung diseases in central Denmark. Respir Med.

[CIT0008] McCunney RJ, Morfeld P, Payne S (2009). What component of coal causes coal workers’ pneumoconiosis?. J Occup Environ Med.

[CIT0009] Yucesoy B, Luster MI (2007). Genetic susceptibility in pneumoconiosis. Toxicol Lett.

[CIT0010] Montelius J, Torén K, Swedish Criteria Group for Occupational Standards (2014). Scientific Basis for Swedish Occupational Standards XXXIII - N-Methyl-2-pyrrolidone /Crystalline Silica, Quartz /Epichlorohydrin.

[CIT0011] Crawford NP, Bodsworth PL, Hadden GG (1982). A study of apparent anomalies between dust levels and pneumoconiosis at British collieries. Ann Occup Hyg.

[CIT0012] Huang X, Li W, Attfield MD (2005). Mapping and prediction of coal workers’ pneumoconiosis with bioavailable iron content in the bituminous coals. Environ Health Perspect.

[CIT0013] Moher D, Liberati A, Tetzlaff J, PRISMA Group (2010). Preferred reporting items for systematic reviews and meta-analyses: the PRISMA statement. Int J Surg.

[CIT0014] Moher D, Cook DJ, Eastwood S (1999). Improving the quality of reports of meta-analyses of randomised controlled trials: the QUOROM statement. Quality of reporting of meta-analyses. Lancet.

[CIT0015] Casswell C, Bergman I, Rossiter CE (1970). The relation of radiological appearance in simple pneumoconiosis of coal workers to the content and composition of the lung. Inhaled Part.

[CIT0016] Seaton A, Dodgson JA, Dick J (1981). Quartz and pneumoconiosis in coalminers. Lancet.

[CIT0017] Hurley JF, Burns J, Copland L (1982). Coalworkers’ simple pneumoconiosis and exposure to dust at 10 British coalmines. Br J Ind Med.

[CIT0018] Jacobsen M, Maclaren WM (1982). Unusual pulmonary observations and exposure to coal mine dust: a case-control study. Ann Occup Hyg.

[CIT0019] Ruckley VA, Fernie JM, Chapman JS (1984). Comparison of radiographic appearances with associated pathology and lung dust content in a group of coalworkers. Br J Ind Med.

[CIT0020] Douglas AN, Robertson A, Chapman JS (1986). Dust exposure, dust recovered from the lung, and associated pathology in a group of British coalminers. Br J Ind Med.

[CIT0021] Morfeld P, Vautrin H, Kösters BA (1997). Components of coal mine dust exposure and the occurrence of prestages of pneumoconiosis. Appl Occup Environ Hyg.

[CIT0022] Buchanan D, Miller BG, Soutar CA (2003). Quantitative relations between exposure to respirable quartz and risk of silicosis. Occup Environ Med.

[CIT0023] Graber JM, Stayner LT, Cohen RA (2014). Respiratory disease mortality among US coal miners; results after 37 years of follow-up. Occup Environ Med.

[CIT0024] Miller BG, Hagen S, Love RG (1998). Risks of silicosis in coalworkers exposed to unusual concentrations of respirable quartz. Occup Environ Med.

[CIT0025] Morfeld P, Noll B, Büchte SF (2010). Effect of dust exposure and nitrogen oxides on lung function parameters of German coalminers: a longitudinal study applying GEE regression 1974-1998. Int Arch Occup Environ Health.

[CIT0026] International Labour Organization (ILO) (2011). Guidelines for the use of the ILO International Classification of Radiographs of Pneumoconioses.

[CIT0027] Heppleston AG (1988). Prevalence and pathogenesis of pneumoconiosis in coal workers. Environ Health Perspect.

[CIT0028] Hurley JF, Alexander WP, Hazledine DJ (1987). Exposure to respirable coalmine dust and incidence of progressive massive fibrosis. Br J Ind Med.

[CIT0029] Maclaren WM, Hurley JF, Collins HP (1989). Factors associated with the development of progressive massive fibrosis in British coalminers: a case-control study. Br J Ind Med.

[CIT0030] Maclaren WM, Soutar CA (1985). Progressive massive fibrosis and simple pneumoconiosis in ex-miners. Br J Ind Med.

[CIT0031] Reisner MT, Bruch J, Hilscher W (1982). Specific harmfulness of respirable dusts from West German coal mines. VI: comparison of experimental and epidemiological results. Ann Occup Hyg.

[CIT0032] Rivers D, Wise ME, King EJ (1960). Dust content, radiology, and pathology in simple pneumoconiosis of coalworkers. Br J Ind Med.

[CIT0033] Jacobsen M (1980). New data on the relationship between simple pneumoconiosis and exposure to coal mine dust. Chest.

[CIT0034] Martin JC, Daniel-Moussard H, Le Bouffant L (1972). The role of quartz in the development of coal workers’ pneumoconiosis. Ann N Y Acad Sci.

[CIT0035] Carta P, Aru G, Barbieri MT (1996). Dust exposure, respiratory symptoms, and longitudinal decline of lung function in young coal miners. Occup Environ Med.

[CIT0036] Ross HF, King EJ, Yoganathan M (1962). Inhalation experiments with coal dust containing 5 per cent, 10 per cent, 20 per cent and 40 per cent quartz: tissue reactions in the lungs of rats. Ann Occup Hyg.

[CIT0037] Peat JK, Salome CM, Xuan W (1996). On adjusting measurements of airway responsiveness for lung size and airway caliber. Am J Respir Crit Care Med.

[CIT0038] Macsali F, Real FG, Plana E (2011). Early age at menarche, lung function, and adult asthma. Am J Respir Crit Care Med.

[CIT0039] Juni P, Holenstein F, Sterne J (2002). Direction and impact of language bias in meta-analyses of controlled trials: empirical study. Int J Epidemiol.

[CIT0040] Travis WD, Costabel U, Hansell DM (2013). An official American Thoracic Society/European Respiratory Society statement: update of the international multidisciplinary classification of the idiopathic interstitial pneumonias. Am J Respir Crit Care Med.

[CIT0041] Baumgartner KB, Samet JM, Stidley CA (1997). Cigarette smoking: a risk factor for idiopathic pulmonary fibrosis. Am J Respir Crit Care Med.

[CIT0042] Cherry NM, Burgess GL, Turner S (1998). Crystalline silica and risk of lung cancer in the potteries. Occup Environ Med.

[CIT0043] Laney AS, Weissman DN (2012). The classic pneumoconioses: new epidemiological and laboratory observations. Clin Chest Med.

[CIT0044] Vallyathan V, Brower PS, Green FH (1996). Radiographic and pathologic correlation of coal workers’ pneumoconiosis. Am J Respir Crit Care Med.

[CIT0045] Castranova V, Vallyathan V (2000). Silicosis and coal workers’ pneumoconiosis. Environ Health Perspect.

[CIT0046] Schins RP, Borm PJ (1999). Mechanisms and mediators in coal dust induced toxicity: a review. Ann Occup Hyg.

[CIT0047] Dalal NS, Newman J, Pack D (1995). Hydroxyl radical generation by coal mine dust: possible implication to coal workers’ pneumoconiosis (CWP). Free Radic Biol Med.

[CIT0048] Dalal NS, Jafari B, Petersen M (1991). Presence of stable coal radicals in autopsied coal miners’ lungs and its possible correlation to coal workers’ pneumoconiosis. Arch Environ Health.

[CIT0049] Ortmeyer CE, Baier EJ, Crawford GM (1973). Life expectancy of Pennsylvania coal miners compensated for disability as affected by pneumoconiosis and ventilatory impairment. Arch Environ Health.

[CIT0050] Bennett JG, Dick JA, Kaplan YS (1979). The relationship between coal rank and the prevalence of pneumoconiosis. Br J Ind Med.

[CIT0051] Mo J, Wang L, Au W (2014). Prevalence of coal workers’ pneumoconiosis in China: a systematic analysis of 2001-2011 studies. Int J Hyg Environ Health.

[CIT0052] Heederik D, Attfield M (2000). Characterization of dust exposure for the study of chronic occupational lung disease: a comparison of different exposure assessment strategies. Am J Epidemiol.

