# Supplementary material for: A systematic review of occupational exposure to coal dust and the risk of interstitial lung diseases
Source: Eur Clin Respir J. 2017 Jan 3;4(1):1264711. doi: 10.1080/20018525.2017.1264711 (PMC5328367; doi:10.1080/20018525.2017.1264711)
Supplement: Supplementary Material [file zecr_a_1264711_sm6183.docx]

**Supplementary material for the paper:**

A systematic review of occupational exposure to coal dust and the risk of interstitial lung diseases

**Table A. Steps in a systematic literature search in Pubmed, EMBASE, Cochrane, Biblioteket.dk and SveMed up to April 2014 on coal exposure and Interstitial lung diseases.**

|  | Papers excluded | Papers left |
| --- | --- | --- |
| Screening article titles (relevant exposure and outcome) | 1878 | 787 |
| Screening article abstract (relevant design, exposure and outcome) | 562 | 225 |
| Reading full paper (all inclusion criteria fulfilled: relevant design, exposure and outcome; sufficient diagnostic criteria, exposure contrast or external control group, one study pr. Dataset, quarts content taken into consideration) | 217 | 8 |
|  | Papers included |  |
| Snow ball search in reviews and included papers | 1 | 9 |


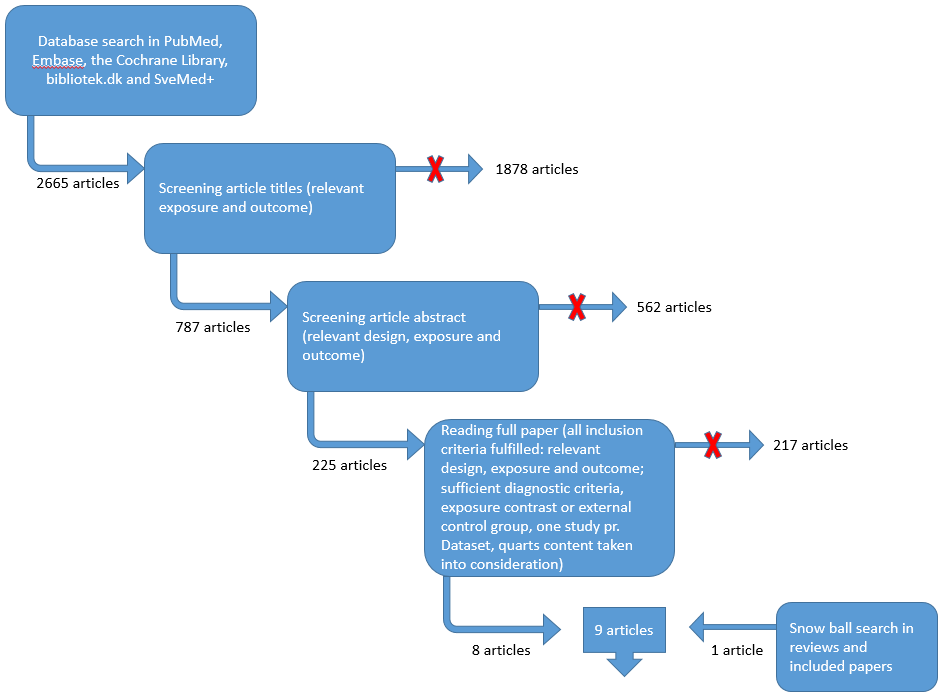


**Figure A:** Flow chart of steps in a systematic literature search in Pubmed, EMBASE, Cochrane, Biblioteket.dk and SveMed up to April 2014 on coal exposure and Interstitial lung diseases.

PubMed, 2255 articles identified. Free text search

## Search terms: Exposure: coal, coal worker, coal miner, coal loading, coal shipping, coal power plant, coal CHP, coal combined heat and power, coal heating plant, coal dust (all fields); Outcome: anthracosis, anthracosilicosis, anthracosis, black lung, anthracosis pneumoconiosis, coal worker pneumoconiosis, coal worker’s pneumoconiosis, coal miners lung, black lung disease, coalworkers pneumoconiosis, coal mine dust lung disease, silicosis) OR pneumoconiosis, bronchial anthracofibrosis, pneumofibrosis, lung fibrosis, pulmonary fibrosis, pulmonary fibrosis, fibrosing alveolitis, fibrosing alveolitides, hamman rich syndrome, interstitial lung disease, parenchymal lung diseases

## Embase, 377 articles identified. MESH term search

Search terms: coal, coal worker, coal miner, coal loading, coal shipping, anthracosis, anthracosilicosis, coal, worker pneumoconiosis, pneumoconiosis, black lung, silicosis, bronchial anthracofibrosis, pneumofibrosis, lung fibrosis, pulmonary fibrosis, fibrosing alveolitis, hamman rich syndrome, parenchymal lung diseases, parenchyma lung diseases, interstitial lung disease

## The Cochrane Library, 249 articles included. Free text search

Search terms: coal

## bibliotek.dk, 54 articles included. Free text search

Search terms: kul* (free text) and lunge* (free text)

## SveMed+, 10 articles included. Free text search

Search terms: coal (free text search), coal, kul, kuleksponering
